# A Novel Approach for Preparing Sepiolite Micron Powder Based on Steam Pressure Changes

**DOI:** 10.3390/ma17143574

**Published:** 2024-07-19

**Authors:** Wenjia Yang, Youhang Zhou, Jialin Song, Yuze Li, Tianyu Gong

**Affiliations:** 1 College of Mechanical Engineering and Mechanics, Xiangtan University, Xiangtan 411105, China; 202131540201@smail.xtu.edu.cn (W.Y.); jlsmi90@126.com (J.S.); yuzeli931@gmail.com (Y.L.); 202231570221@smail.xtu.edu.cn (T.G.); 2 Engineering Research Center of Complex Tracks Processing Technology and Equipment of Ministry of Education, Xiangtan University, Xiangtan 411105, China

**Keywords:** sepiolite, powder preparation, steam pressure change, morphological characterization, specific surface area

## Abstract

As a common method for preparing micron powder in industrial operations, the mechanical extrusion method simply pursues the particle size without considering the microstructure characteristics of sepiolite, which leads to problems such as bundles of sepiolite not being effectively dispersed, and thus the disruption of fibers is inevitably caused. In this work, a new micronization method for disaggregating these bundles while preserving the original structural integrity of the fibers is proposed based on steam pressure changes. The effects of steam pressure changes on the particle size distribution, microstructure, and properties of treated sepiolite are studied using X-ray fluorescence spectrometer (XRF), X-ray diffractometer (XRD), Field Emission Scanning Electron Microscopy (FESEM), Transmission Electron Microscopy (TEM), and a specific surface area and aperture analyzer (BET). The experimental results show that the particle size of sepiolite powder depends greatly on steam pressure, and sepiolite powder with mass ratio of 91.6% and a particle size D97 of 21.27 μm is obtained at a steam pressure of 0.6 MPa. Compared to the sepiolite after mechanical extrusion, the sepiolite treated with steam pressure changes can maintain the integrity of its crystalline structure. The specific surface area of sepiolite enhanced from 80.15 m^2^ g^−1^ to 141.63 m^2^ g^−1^ as the steam pressure increased from 0.1 to 0.6 MPa, which is about 1.6 times that of the sample treated with mechanical extrusion.

## 1. Introduction

Sepiolite is a kind of water-bearing magnesium silicate clay. Due to its naturally occurred nanometer crystalline structure with a large aspect ratio, it exhibits good adsorption, catalytic properties, and high-temperature resistance [1,2,3]. Sepiolite micron powder is widely used in the chemical industry [4,5,6,7], building materials [8,9,10], medicine [11,12,13,14], and other fields. At present, in the sepiolite powder processing industry, the mechanical extrusion method is widely used to prepare micron powder. Although this method can be used to prepare micron powders with a uniform particle size distribution on a large scale, the bundles of sepiolite are not effectively dispersed due to the dual effects of uncontrollable extrusion force and shear force over a long time. Moreover, this inevitably causes the disruption of fibers, resulting in a decrease in the aspect ratio [15,16]. The mechanical extrusion method, which simply pursues the particle size and ignores the microstructural characteristics of the sepiolite, is obviously not optimal. Therefore, based on the microstructural characteristics of sepiolite, a new micronization method should be introduced to disaggregate the bundles while preserving the original structural integrity of the fibers.

Researchers [17,18,19,20] have found that clays such as sepiolite and palygorskite contain many natural pores observed under SEM. These pores can be described as intracrystalline or structural micropores and textural pores (inter-fiber micropores and mesopores, macropores formed by aggregation of bundles) [21,22]. Due to the high level of water adsorption that clay is capable of, all of its pores are filled with adsorbed water (H_2_O) in a humid environment [23,24]. Based on the microstructure mentioned above, researchers have proposed a new green technology, named the freezing method, in the study of palygorskite aggregate dispersion [25,26]. It has been reported that when palygorskite was frozen at low temperature, the adsorbed water in the textural pores turned to ice and the volume increased by about 10 times. This phenomenon can cause the pores to swell and disperse the bundles into multiple smaller-sized forms, and the aspect ratio of the fibers was kept intact. However, the pores’ volume increase after this freezing treatment was limited. This may lead to incomplete disaggregation, resulting in an unsatisfactory splintering effect. Theoretically, it has been found that liquid water will turn into steam at temperatures above 100 °C, and its volume will theoretically increase by about 1000 times [27]. In addition, in the range of 100–300 °C, the adsorbed water in the pores will change from a liquid phase to a gas phase, but the crystalline structure will not be destroyed [28,29]. Accordingly, if sepiolite is placed in a pressure vessel filled with high-temperature steam, its pore volume will increase significantly. Meanwhile, stress concentration occurs at the edge of the pores, causing the aggregates to initiate dispersion.

Similar to the freezing method, if only considering the state change of adsorbed water in the pores with the aim of splintering sepiolite, the effect achieved with the steam method may not be ideal. To allow the sepiolite to become completely splintered, new energy must be added. Moreover, the pressure vessel contains steam, and its pressure is higher than atmospheric pressure, so further research can be carried out using these two objective conditions. Some scholars have carried out related research on crack extension under pressure changes. In terms of crack extension, pre-cracked eggs were placed in a customized pressure chamber, and then it was observed through the equipment that when the pressure suddenly decreased, the eggshell expanded instantly, resulting in rapid crack extension [30]. The crack extension process of soft rock subjected to confining pressure during unloading pressure was also studied [31]. It was found that as the unloading rate of external confining pressure increases, the brittleness characteristics of soft rock become more obvious, and the rate of crack extension also increases. Based on the above studies, under the premise of ensuring safety, a pressure relief device can be used to instantly unload the pressure inside a pressure vessel, and the resulting pressure change can be applied to splinter sepiolite.

In this work, a new, simple, and efficient method for splintering sepiolite using steam pressure changes is adopted. Six groups of powder preparation experiments under different steam pressure values and one powder preparation experiment using mechanical extrusion were carried out for comparative study. The effects of steam pressure changes and mechanical extrusion processes on the particle size distribution, XRD patterns, morphology, and microstructure of sepiolite are thus investigated systematically.

## 2. Materials and Methods

### 2.1. Material

**Figure 1:**
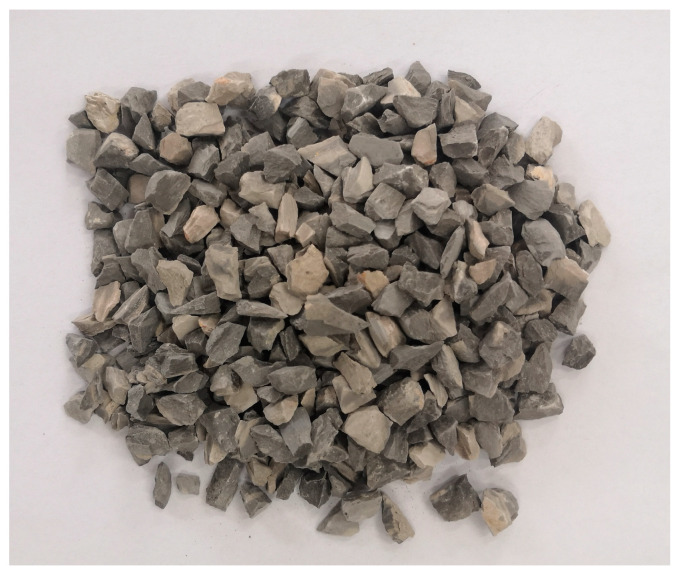
Sepiolite sample.

Natural sepiolite mineral (Figure 1) was obtained from Xiangtan, China. A chemical composition analysis of this sample (Table 1) was accomplished with an X-ray Fluorescence Spectrometer (XRF, DF-1000, Shenzhen Cepu Technology Co., Ltd., Shenzhen, China).

### 2.2. Experimental Method

**Figure 2:**
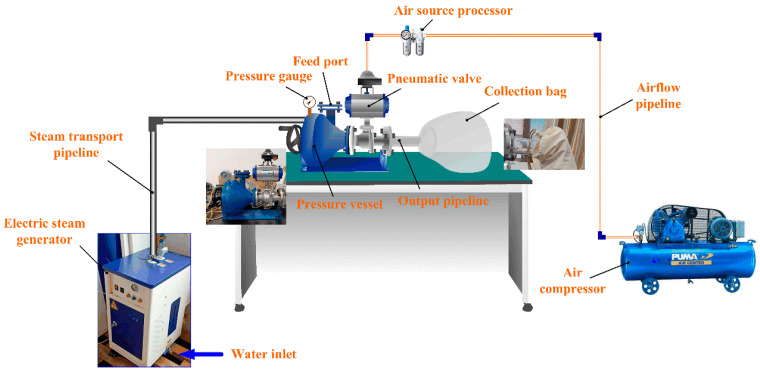
Experimental platform.

**Figure 3:**
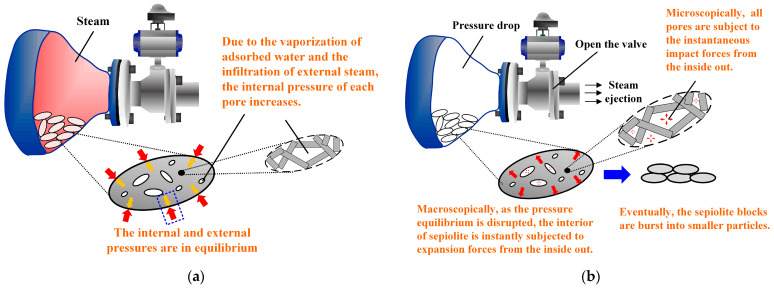
The principle of preparing sepiolite powder using this method: (**a**) Steam injection. (**b**) Steam release.

The experimental platform is shown in Figure 2. In this study, a total of 6 experiments were carried out according to the set pressure values (Table 2) in the pressure vessel. Specifically, a pre-prepared 60 g sepiolite sample (particle size smaller than 2 cm) was put into a pressure vessel, and then high-temperature steam was injected into the vessel via an electric steam generator (rated working pressure of 0.7 MPa, rated saturated-steam temperature of 171 °C). When the pressure inside the pressure vessel reached the set value, the pressure was instantly unloaded through a pneumatic valve. Finally, the sepiolite powder in the pressure vessel flowed into the collection bag through the output pipeline. The principle of preparing sepiolite powder using this method is shown in Figure 3. The samples were denoted as SEP-0.1, SEP-0.2, SEP-0.3, SEP-0.4, SEP-0.5, and SEP-0.6 according to the pressure in the vessel. The sample (SEP-ext) prepared with mechanical extrusion (XPF175, Jiangxi Weiming Machinery Equipment Co., Ltd., Ganzhou, China) was used as the contrast sample to analyze the effects of the proposed method on the fiber structure and adsorption property of sepiolite.

### 2.3. Characterization

XRD patterns were collected from 3 to 50° (2θ) using an X-ray diffractometer (Smart lab, Rigaku, Tokyo, Japan). The morphology of the samples was observed using Field Emission Scanning Electron Microscopy (FESEM, SU5000, HITACHI, Tokyo, Japan) and Transmission Electron Microscopy (TEM, Talos F200S, Thermo Scientific, Waltham, MA, USA). Before the FESEM observation, all samples needed to be coated with gold. The preparation procedure for the TEM sample was as follows: a drop of the diluted suspension was deposited on a microscopic grid with collodion; N_2_ adsorption–desorption isotherms were obtained using a specific surface area and aperture analyzer at 77 K (SSA4000, Beijing Builder Electronic Technology Co., Ltd., Beijing, China). Since sepiolite contains micropores inside, all samples needed to be preheated at 100° for 12 h under N_2_ to remove the adsorbed water. The particle size distributions of the samples were measured using a laser particle size analyzer (LAP-W800H, Xiamen Yishite Instruments Co., Ltd., Xiamen, China).

## 3. Results and Discussion

### 3.1. Particle Size Distribution

**Figure 4:**
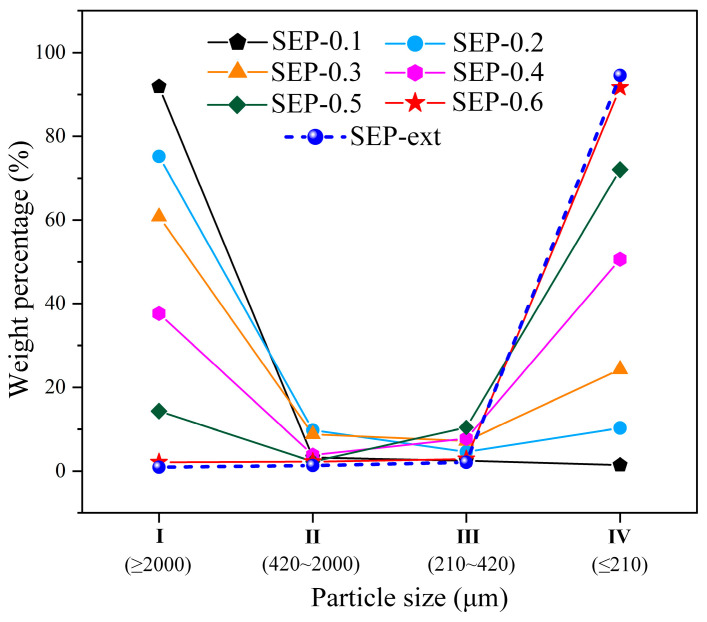
Distribution of the proportions of mass among the samples.

The sepiolite powders obtained in the experiment were classified according to particle size by using standard sieves. The particle size distribution of the samples is shown in Figure 4. When the pressure values were 0.1~0.3 MPa, the weight percentages of powder in classification I (d ≥ 2000 μm) reached more than 60%, and the weight percentages of powder in classification IV (d ≤ 210 μm) did not exceed 25%. This indicates that the sepiolite splintering effect is not ideal under these pressure values. When pressure values were 0.4~0.6 MPa, the weight percentages of powder in classification I decreased significantly to 37.6%, 14.3%, and 2.1%, respectively, and the weight percentages of powder in classification IV increased significantly to 50.6%, 72.1%, and 91.6%, respectively. It is thus proven that this method can make a sample of sepiolite split into powder with a smaller particle size. The greater the steam pressure, the higher the amount of powder obtained with a smaller particle size. In addition, Figure 4 shows that for both samples SEP-0.6 and SEP-ext, the weight percentage of powder with the particle size of classification IV (d ≤ 210 μm) was more than 90%, indicating that sepiolite powder can be prepared effectively using the method proposed in this paper.

**Figure 5:**
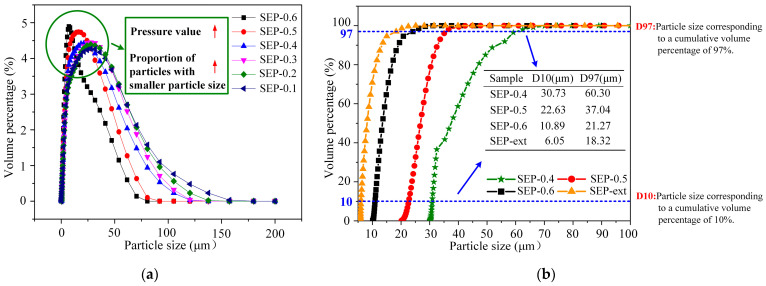
Particle size distribution results: (**a**) Particle size distribution of samples treated with steam (≤210 μm). (**b**) Cumulative particle size distribution of all samples.

Considering the limitation of standard sieve measurement, a laser particle size analyzer was used to accurately measure the particle size distribution of the sepiolite powders (d ≤ 210 μm). As shown in Figure 5a, with the increase in steam pressure, the proportion of sepiolite powders with particle sizes ranging from 0 to 50 μm gradually increased. In the powder processing industry, D10 and D97 are two important indexes for evaluating powder size in powder production and application. The D97 values of the samples SEP-0.4, SEP-0.5, and SEP-0.6 (Figure 5b) are 60.30, 37.04, and 21.27 μm, respectively, indicating that the particle sizes of powders greatly decreased as the steam pressures increased. The gaps between the D97 and D10 values of samples SEP-0.4, SEP-0.5, and SEP-0.6 are 29.57, 14.41, and 10.38 μm, respectively. Obviously, the higher the steam pressure value, the more concentrated the particle size distribution of the powder. In general, compared to the sample SEP-ext, although the particle size of sample SEP-0.6 is slightly larger, the distribution is more concentrated, which indicates that the method proposed in this paper has potential for applications in the powder processing industry.

### 3.2. Morphological Characterization

Compared to mechanical extrusion, the steam pressure change process could introduce volume expansion without shearing force being applied to the textural pores of sepiolite, which would disaggregate the crystal bundles and splinter the sepiolite.

**Figure 6:**
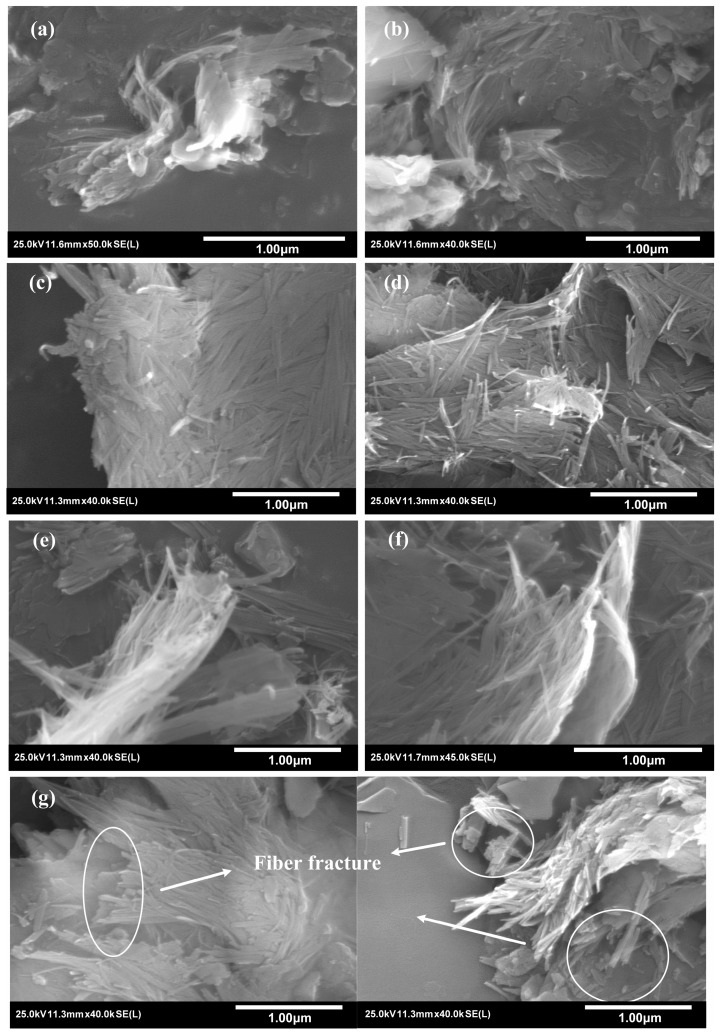
SEM images of samples: (**a**) SEP-0.1, (**b**) SEP-0.2, (**c**) SEP-0.3, (**d**) SEP-0.4, (**e**) SEP-0.5, (**f**) SEP-0.6, (**g**) SEP-ext.

To reveal the changes in the morphological characteristics of sepiolite achieved with steam pressure changes and mechanical extrusion, the SEM images of the samples are discussed here. In the SEP-0.1 and SEP-0.2 samples (Figure 6a,b), the sepiolite appears as a tightly bound aggregate, and the fibers are barely visible. In the SEP-0.3 sample (Figure 6c), large bundles formed by tightly bound fibers are observed, and no fibers are found to be dispersed. In the SEP-0.4 sample (Figure 6d), fluffy bundles and rod-like fibers dispersed from these bundles can be observed in a few areas. In the SEP-0.5 (Figure 6e) and SEP-0.6 (Figure 6f) samples, a larger range of fluffy bundles can be clearly observed. The above results show that with the increase in pressure, the tightly agglomerated bundles gradually become fluffy, and the textural porosity (inter-fiber microporosity and mesoporosity) may gradually increase. However, in the SEP-ext sample, the sepiolite still appears to be a tightly bound aggregate, the bundles are not fluffy, and some fibers with a shorter length are observed, which could have been caused by the disruption of original fibers (Figure 6g).

**Figure 7:**
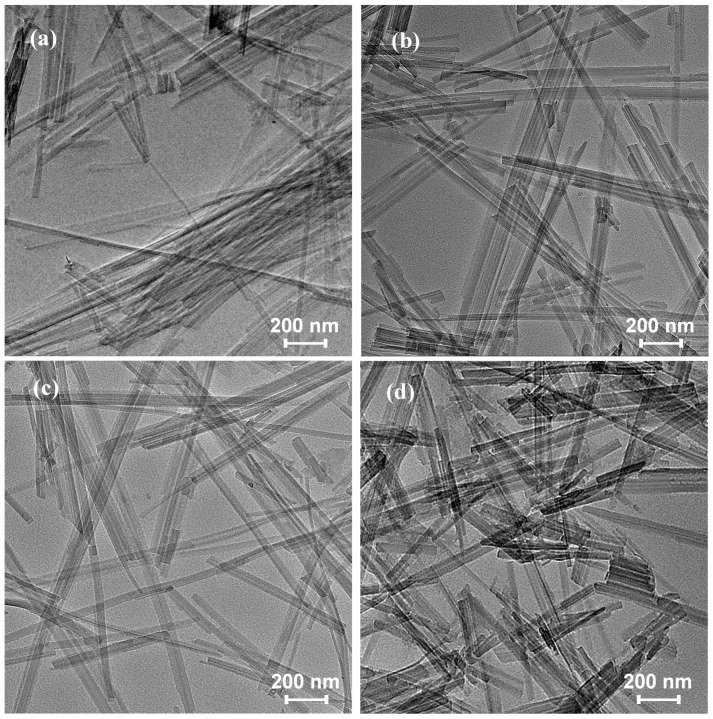
TEM images of samples: (**a**) SEP-0.4, (**b**) SEP-0.5, (**c**) SEP-0.6, (**d**) SEP-ext.

To further evaluate the effect of these treatment methods on sepiolite, we observe the TEM images of the SEP-0.4, SEP-0.5, SEP-0.6, and SEP-ext samples shown in Figure 7. The length ranges of the fibers were estimated using Image J software (Image J 1.43c) from the TEM images. In the SEP-0.4 sample (Figure 7a), a small number of single fibers and bundles with a large diameter formed by many parallel fibers are observed. Most of these fibers are 1–2 μm in length [3,32]. As the pressure increases (SEP-0.5 and SEP-0.6), more successfully disaggregated fibers are observed, and the large bundles are also changed into smaller bundles formed by 2–8 parallel fibers. The fiber length is basically unchanged (Figure 7b,c). In the SEP-ext sample (Figure 7d), some bundles formed by parallel fibers with lengths of 110 nm–590 nm are observed. This indicates that the bundles were not effectively disaggregated when this sample was treated with mechanical extrusion. Meanwhile, a disruption of the original fibers occurred. This conclusion is supported by the results of the aforementioned SEM observation.

### 3.3. XRD Analysis

**Figure 8:**
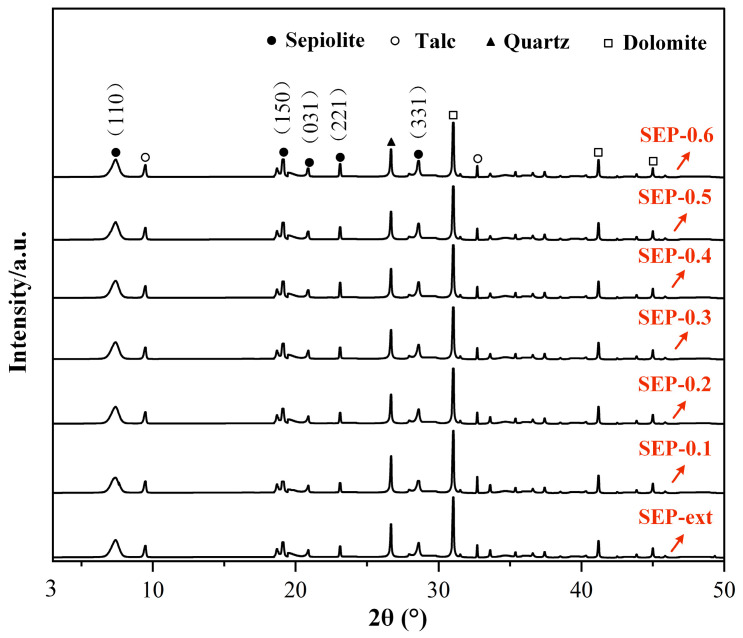
XRD patterns of the samples.

To explore the effects of these treatment methods on the sepiolite’s crystalline structure, an XRD analysis is carried out. As shown in Figure 8, the characteristic peaks of sepiolite are the diffraction peaks at 2θ = 7.38°, 19.06°, 20.87°, 23.11°, and 28.61°, respectively. The characteristic peaks of talc impurity are the diffraction peaks at 2θ = 9.46° and 32.69°. The characteristic peak of quartz impurity is the diffraction peak at 2θ = 26.67°. The characteristic peaks of dolomite impurity are the diffraction peaks at 2θ = 31.01°, 41.17°, and 44.98°, respectively. The XRD pattern results show that the intensity and position of each diffraction peak of sepiolite were almost unchanged when the sepiolite was treated with steam (Figure 8). This indicates that steam pressure change process has no effect on the sepiolite crystalline structure.

### 3.4. Microstructure Analysis

**Figure 9:**
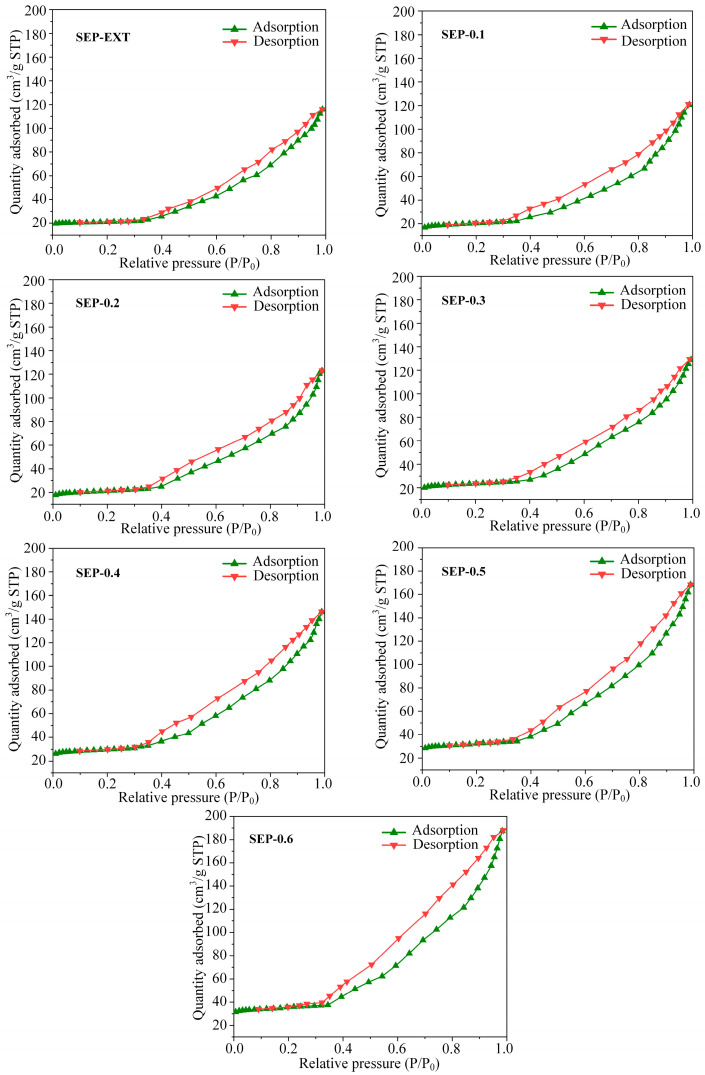
N_2_ adsorption–desorption isotherms of the samples.

Adsorptiveness is one of the important properties that determines the application value of sepiolite, and it is mainly evaluated using microstructural parameters, especially the specific surface area [33,34]. Based on the analysis of its effect on the morphological characteristics of sepiolite, it can be found that the steam pressure change treatment has an influence on the microporosity of sepiolite, as shown in Figure 6. Therefore, it is necessary to analyze the effect of steam pressure change treatment on the microstructure parameters of sepiolite. Figure 9 shows that the nitrogen adsorption–desorption curves of sepiolite belong to a type IV isotherm containing an H3-type hysteresis loop. This indicates that the internal pore structure of sepiolite is very irregular. Moreover, with the increase in the pressure, the adsorption capacity of the samples continues to increase in the range of relative pressure P/P_0_ > 0.9. This may have been caused by the gradual increase in large pores inside the sepiolite.

Table 3 lists the microstructural parameters of the samples. The values of specific surface area (*S*_BET_) were calculated with the BET method. The t-plot method was used to estimate the micropore volume (*V*_micro_). The *S*_BET_ values of SEP-ext, SEP-0.1, and SEP-0.2 are 88.73, 80.15, and 84.96 m^2^ g^−1^, respectively, indicating that low steam pressure levels could not improve the textural microporosity of sepiolite. This postulate is further supported by the low microporosity of SEP-0.1 and SEP-0.2 shown in Table 3. In addition, due to the fiber length of the SEP-ext sample being shorter, the *S*_BET_ of SEP-ext is higher when the porosity is similar [22]. The higher steam pressure significantly increased the *S*_BET_ of the sepiolite. Specifically, the *S*_BET_ of SEP-0.6 displays a maximum of 141.63 m^2^ g^−1^. Similar to the change in *S*_BET_, the *S*_micro_ and *V*_micro_ values also significantly increased from 15.81 m^2^ g^−1^ (0.1 MPa) to 97.66 m^2^ g^−1^ (0.6 MPa) and from 0.0047 cm^3^ g^−1^ (0.1 MPa) to 0.0152 cm^3^ g^−1^ (0.6 MPa), respectively. These results indicate that the difference between the pressure inside the textural pores and that outside the textural pores during the steam pressure change process could expand the gaps among the fibers inside the bundles. As the pressure increases, the bundles become more fluffy, thus improving accessibility to the external and textural microporosity. This conclusion can be supported by the results of the SEM observation.

## 4. Conclusions

From the data reported above, the following conclusions can be drawn:

1. Sepiolite powders with a mass ratio of more than 90% and a particle size of less than 22 μm can be successfully obtained at the steam pressure of 0.6 MPa.
2. The steam pressure change method has obvious advantages over mechanical extrusion in preserving fiber integrity.
3. Compared to the sepiolite after extrusion, the specific surface area and micropore volume of the sepiolite subjected to steam pressure changes were increased by 59.6% and 216.7%, respectively. This indicates that the steam pressure change method could be described as a method for activating the surface of sepiolite. The sepiolite micron powders prepared using this method have a certain potential for applications as adsorbents.

Due to the limited experimental conditions, an experiment with higher pressure values could not be carried out in this study. In a future study, an experimental device with higher safety performance should be designed to carry out an experiment with higher pressure values, so that more ideal experimental results could be obtained.

## Figures and Tables

**Table 1:** Chemical composition analysis of the sepiolite sample.

Constituent	Weight %
SiO_2_	52.79
MgO	17.32
Al_2_O_3_	3.21
CaO	3.37
Fe_2_O_3_	0.75
Na_2_O	0.08
K_2_O	0.91
TiO_2_	0.25
Loss on ignition (LOI)	21.32

**Table 2:** The relationship between steam pressure and temperature in the experiments.

Sample	Pressure (MPa)	Temperature (°C)
SEP-0.1	0.1	99.09
SEP-0.2	0.2	119.62
SEP-0.3	0.3	132.68
SEP-0.4	0.4	142.92
SEP-0.5	0.5	151.11
SEP-0.6	0.6	158.08

**Table 3:** Specific surface area (*S*_BET_), micropore area (*S*_micro_), micropore volume (*V*_micro_), and total pore volume (*V*_total_) of the samples.

Sample	*S*_BET_ (m^2^ g^−1^)	*S*_micro_ (m^2^ g^−1^)	*V*_micro_ (cm^3^ g^−1^)	*V*_total_ (cm^3^ g^−1^)
SEP-ext	88.73	16.76	0.0048	0.1841
SEP-0.1	80.15	15.81	0.0047	0.1773
SEP-0.2	84.96	17.51	0.0049	0.1963
SEP-0.3	93.25	32.58	0.0069	0.2438
SEP-0.4	111.76	61.13	0.0115	0.2520
SEP-0.5	124.05	80.39	0.0120	0.2573
SEP-0.6	141.63	97.66	0.0152	0.2681

## Data Availability

The original contributions presented in the study are included in the article, further inquiries can be directed to the corresponding author.
